# Promoter Variants of the ADAM10 Gene and Their Roles in Temporal Lobe Epilepsy

**DOI:** 10.3389/fneur.2016.00108

**Published:** 2016-06-30

**Authors:** Hua Tao, Jianghao Zhao, Xu Zhou, Zhonghua Ma, Ying Chen, Fuhai Sun, Lili Cui, Haihong Zhou, Yujie Cai, Yanyan Chen, Shu Zhao, Lifen Yao, Bin Zhao, Keshen Li

**Affiliations:** ^1^Department of Neurology, Affiliated Hospital of Guangdong Medical University, Zhanjiang, Guangdong, China; ^2^Guangdong Key Laboratory of Age-related Cardiac and Cerebral Diseases, Guangdong Medical University, Zhanjiang, Guangdong, China; ^3^Clinical Research Center, Guangdong Medical University, Zhanjiang, Guangdong, China; ^4^Department of Neurology, Beijing Tongren Hospital, Capital Medical University, Beijing, China; ^5^Department of Neurology, Central People’s Hospital of Zhanjiang, Zhanjiang, Guangdong, China; ^6^Department of Neurology, The First People’s Hospital of Pingdingshan, Pingdingshan, Hebei, China; ^7^Department of Neurology, The First Affiliated Hospital of Harbin Medical University, Harbin, Heilongjiang, China; ^8^Jinan University, Guangzhou, Guangdong, China

**Keywords:** ADAM10, promoter variants, single nucleotide polymorphisms, epileptic seizures, temporal lobe epilepsy

## Abstract

Previous evidence has indicated that downregulated ADAM10 gives rise to epileptic seizures in Alzheimer’s disease, and this study investigated the association of ADAM10 with temporal lobe epilepsy (TLE) from a genetic perspective. A total of 496 TLE patients and 528 healthy individuals were enrolled and genotyped for ADAM10 promoter variants (rs653765 G > A and rs514049 A > C). The alleles, genotypes, and haplotypes were then compared with clarify the association of these variants with TLE and their impacts upon age at onset, initial seizure types before treatments, and responses to drug treatments. In cohorts I, II, and I + II, the frequencies of the A allele and AA genotype at rs514049 were consistently increased in the cases compared with the controls (*p* = 0.020 and *p* = 0.009; *p* = 0.008 and *p* = 0.009; *p* = 0.000 and *p* = 0.000; *q* = 0.003 and *q* = 0.002, respectively). In contrast, the frequency of the AC haplotype (rs653765–rs514049) decreased in cohorts I + II (*p* = 0.013). Further analyses of the TLE patients indicated that the AA genotype functioned as a predisposing factor to drug-resistant TLE and the AC haplotype as a protective factor against generalized tonic–clonic seizures (GTCS) and drug-resistant TLE. This study is the first to demonstrate an association of the ADAM10 promoter variants with TLE. In particular, the AA genotype and AC haplotype showed their effects upon GTCS and drug-resistant TLE.

## Introduction

The past decades have witnessed great efforts to investigate temporal lobe epilepsy (TLE), but its pathogenesis remains unclear (1). Notably, some brain diseases are associated with increased risks of seizures, especially Alzheimer’s disease (AD), with a 6- to 10-fold risk of developing seizures (2–8). Interestingly, AD usually undergoes abnormal alterations in temporal lobes, whereas TLE experiences a certain degree of cognitive dysfunction, suggesting that these two common diseases may share underlying mechanisms. Hence, further exploration of key modulators in AD may represent a novel way to understand TLE.

Indeed, the deposition of amyloid β (Aβ), a pathological hallmark of AD, has been considered a probable link to epileptic seizures by increased neuronal destruction in response to excitotoxicity (9, 10). ADAM10, a key α-secretase for amyloid precursor protein (APP), functions in the formation of soluble APP alpha (sAPPα), which is a competitive biological process that inhibits the cleavage of APP from the overproduction of neurotoxic Aβ (11). Strikingly, the levels of ADAM10 decrease in the pyramidal cell layers of the rat hippocampus after status epilepticus (12) and in the brain tissues of AD patients (13). After the gene encoding ADAM10 was knocked out in mice, the expression level of ADAM10 was downregulated in the brain cortex; this was accompanied by repetitive seizures (14), which indicate that insufficient expression of ADAM10 could represent a potential etiology of epileptic seizures in AD patients. Further research using a transgenic mouse model of experimental kainate-induced seizures uncovered the detailed role of ADAM10, and mice with moderate overexpression of ADAM10 suffered from weaker seizures with a shorter recovery period in comparison to mice with low expression of ADAM10 (15). Hence, ADAM10 should play a protective role against epileptic activities.

In light of the single nucleotide polymorphism database (dbSNP), 6,771 polymorphic sites exist in the sequence of the ADAM10 gene. In theory, these SNPs might influence predisposition to related diseases by altering the expression of ADAM10 or certain linkage correlations. Among these are rs653765 and rs514049, two variants of great concern, located in the promoter region and 5′ untranslated region (UTR) of the ADAM10 gene, respectively. Previous studies have documented their association with AD (16–19). In particular, Bekris and colleagues found that the AC haplotype (rs653765–rs514049) was associated with a lower level of ADAM10 protein in the postmortem hippocampus of AD patients (17). Moreover, these researchers further observed that sAPPα expression was lower in the cerebrospinal fluid of AD patients with the AC haplotype in comparison to controls with the same haplotype, which is consistent with the insufficient expression of ADAM10 in AD patients. More importantly, the AC haplotype was confirmed to reduce transcriptional activities of the ADAM10 promoter through a dual-luciferase reporter gene assay system (17). Nonetheless, the association of ADAM10 with TLE remains undetermined from the perspective of genetics.

To identify whether the promoter variants (rs653765 and rs514049) function in TLE, this study recruited two independent cohorts from the Han Chinese population, with the goal of clarifying the genetic implications of ADAM10 in the occurrence and development of TLE.

## Materials and Methods

### Ethical Standards

This study was approved by the Ethics Committees of the Affiliated Hospital of Guangdong Medical University, the First Affiliated Hospital of Harbin Medical University, the Beijing Tongren Hospital, the Central People’s Hospital of Zhanjiang, and the First People’s Hospital of Pingdingshan and was performed according to the Declaration of Helsinki. Informed-consent documents were signed by all subjects before their enrollments.

### Subject Enrollment

Initially, 335 TLE patients (male/female: 170/165; mean age: 31 ± 15 years) and 325 healthy individuals (male/female: 185/140; mean age: 31 ± 10 years) were enrolled into cohort I. All of the subjects were recruited from three hospitals (the First Affiliated Hospital of Harbin Medical University, the Affiliated Hospital of Guangdong Medical University, and the Beijing Tongren Hospital). To validate the genotyping findings in cohort I, an additional group of 161 TLE patients (male/female: 79/82; mean age: 33 ± 15 years) and 203 healthy individuals (male/female: 104/99; mean age: 33 ± 16 years) was recruited from the Central People’s Hospital of Zhanjiang and the First People’s Hospital of Pingdingshan as cohort II. All of the subjects in cohorts I + II were Han Chinese and constituted a total of 496 TLE patients and 528 healthy individuals.

The gender, age, age at onset, and initial seizure types [such as complex partial seizures (CPS) and generalized tonic–clonic seizures (GTCS)] before treatments were documented at the time of enrollment. Seizure frequencies were further recorded after their enrollments through field or telephone investigation. According to the definition of drug-resistant epilepsy (DRE) proposed by the International League Against Epilepsy (ILAE) (20), the responses to drug treatments of the TLE patients were defined as follows: drug-resistant cases were determined in line with the absence of a change or a reduction (<60%) in seizure frequency after 1 year of treatment with a schedule of two or more tolerated and appropriately selected antiepileptic drugs; the remaining were considered as drug-sensitive cases. In addition, all subjects whose blood samples failed to be genotyped were excluded from the study.

### DNA Extraction and Genotyping for the ADAM10 SNPs

Peripheral blood samples were collected from each subject. DNA was then extracted using the Genomic DNA Extraction Kit (Tiangen Biotech, Beijing, China) and temporarily stored at −80°C prior to genotyping. The DNA samples were genotyped for the ADAM10 SNPs (rs514049 and rs653765) using the ABI PRISM SNaPshot method (Applied Biosystems, Carlsbad, CA, USA). The primers used in multiplex PCR for amplification of two target fragments (121 and 155 bp, respectively) were as follows: rs514049, 5′-TGAGGACCTTCCCTTGGGCTAA-3′ (forward primer) and 5′-GGTGCACCAAGAGAGGCAGAAA-3′ (reverse primer); rs653765, 5′-CGGCAACGCTCCTAGCTCCT-3′ (forward primer) and 5′-CGCGTCACGTGGTGAGGA-AG-3′ (reverse primer). The primers used in SNaPshot PCR for primer extension were 5′-TTTTTTTTTTTTTTTTTTTTTTTTT AAGCAGGGCTGCTTTCGACTTCTT-AA-3′ (rs514049) and 5′-TTTTTTTTTTTTTTTTTTTTTTTTTTTTTT TTTTTTTTT-GAGGCGGAGGTCTGAGTTTCGA-3′ (rs653765).

The multiplex PCR reaction mix was composed of 1× HotStarTaq buffer, 3.0 mM Mg^2+^, 0.3 mM dNTP, 1 U HotStarTaq polymerase, 1 μl DNA template, and 1 μl primer mix. The PCR program was as follows: an initial cycle at 95°C/2 min; 11 cycles of 94°C/20 s, 65°C/40 s, and 72°C/90 s; 24 cycles of 94°C/20 s, 59°C/30 s, and 72°C/90 s; and a final cycle at 72°C/2 min. The products were then purified with the assistance of Shrimp alkaline phosphatase (SAP) and exonuclease I. The SNaPshot PCR reaction mix contained 5 μl SNaPshot Multiplex Kit, 2 μl purified PCR products, 1 μl primer mix, and 2 μl ultrapure H_2_O. The PCR program was as follows: an initial cycle at 96°C/1 min; 28 cycles of 96°C/10 s, 55°C/5 s, and 60°C/30 s; and a final cycle at 4°C/2 min. After further purification by SAP, the extension products were analyzed using the ABI 3730xl DNA Analyzer and GeneMapper 4.1 (Applied Biosystems, Carlsbad, CA, USA). Finally, 5% of samples were randomly selected for genotyping for quality control in an independent trial. The genotyping results had to be entirely consistent with the primary results, otherwise the remaining (95% of samples) would be genotyped again to find the discordant samples, which would then be excluded from the study.

### Statistical Analyses

The measurement data are shown as the means ± SD and compared using Student’s *t*-test. The enumeration data were compared using Chi-squared test or Fisher’s exact test. Logistic regression was used to correct bias of confounding factors, such as age and gender, and *q* values were calculated using Bonferroni correction for adjusting false positive results in multiple-time statistics. Most statistical analyses were carried out by SPSS 19.0 (IBM, NY, USA), and a two-tailed *p* ≤ 0.05 was considered significant. In addition, power analyses were performed by Quanto 1.2 (University of Southern California, LA, USA) and haplotype analyses by Haploview 4.2 (Daly Lab, Cambridge, MA, USA).

## Results

### Subject Characteristics

In total, this study enrolled 496 TLE patients and 528 healthy individuals. No significant differences in gender or age were observed between the cases and the controls in cohorts I, II, and I + II (all *p* values >0.05). In addition to gender and age, other characteristics of the TLE patients in cohorts I + II, including age at onset, initial seizure types before treatments, and responses to drug treatments, are shown in Table 1.

**Table 1:** **Subject characteristics**.

	Cases	Controls	*p* Values
**Gender (male/female, *n*)**			
Cohort I	170/165	185/140	0.112
Cohort II	79/82	104/99	0.682
Cohorts I + II	249/247	289/239	0.147
**Age (mean ± SD, years)**			
Cohort I	31 ± 15	31 ± 10	0.862
Cohort II	33 ± 15	33 ± 16	0.617
Cohorts I + II	32 ± 15	32 ± 13	0.743
**Other characteristics in cohorts I + II**			
Age at onset (mean ± SD, years)	21 ± 14	–	–
**Initial seizure types before treatment (*n***)			
CPS/non-CPS cases	332/164	–	–
GTCS/non-GTCS cases	184/312	–	–
**Responses to drug treatment**			
Seizure frequency (mean ± SD, times/month)	7 ± 3	–	–
Drug-resistant/sensitive cases (*n*)	246/250	–	–

### Data Evaluations

All samples were successfully genotyped in this study. The frequency distributions of the ADAM10 SNPs (rs653765 and rs514049) in the case and control groups complied with Hardy–Weinberg equilibrium. Power analyses using a log-additive mode showed that this study would have 99.8% power for rs653765 and 90.7% power for rs514049 to detect a genotype with an odds ratio of 1.7 at a significance level of 0.05.

### Frequency Distributions of the ADAM10 SNPs

In cohort I, the frequencies of the A allele and AA genotype at rs514049 A > C significantly increased in the cases compared with the controls (Table 2, *p* = 0.020 and *p* = 0.009, respectively). Moreover, their frequencies simultaneously increased in the cases compared with the controls in cohorts II (Table 2, *p* = 0.008 and *p* = 0.009, respectively) and I + II (Table 3, *p* = 0.003 and *p* = 0.002, respectively), which confirmed the findings in cohort I. These results indicate that the A allele and AA genotype could be associated with predisposition to TLE. However, no significant differences were observed in the ADAM10 SNPs at rs653765 G > A in the study.

**Table 2:** **Alleles and genotypes of the ADAM10 SNPs in the cases and controls of cohorts I and II**.

	Cohort I			Cohort II		
	Cases *n* (%)	Controls *n* (%)	*p* Values	Cases *n* (%)	Controls *n* (%)	*p* Values
**rs653765 G > A**						
G/A	560 (83.6)/110 (16.4)	538 (82.8)/112 (17.2)	0.816	272 (84.5)/50 (15.5)	336 (82.8)/70 (17.2)	0.582
GG/GA/AA	233 (69.6)/94 (28.1)/8 (2.4)	223 (68.6)/92 (28.3)/10 (3.1)	0.922	115 (71.4)/42 (26.1)/4 (2.5)	140 (69.0)/56 (27.6)/7 (3.4)	0.584
GG/GA + AA	233 (69.6)/102 (30.5)	229 (68.6)/102 (31.4)	0.815	115 (71.4)/46 (28.6)	140 (69.0)/63 (31.0)	0.666
GG + GA/AA	327 (97.6)/8 (2.4)	315 (96.9)/10 (3.1)	0.635	157 (97.5)/4 (2.5)	196 (96.6)/7 (3.4)	0.592
**rs514049 A > C**						
A/C	640 (95.5)/30 (4.5)	600 (92.3)/50 (7.7)	0.020	309 (96.0)/13 (4.0)	368 (90.6)/38 (9.4)	0.008
AA/AC/CC	309 (92.2)/22 (6.6)/4 (1.2)	278 (85.5)/44 (13.5)/3 (0.9)	0.100	150 (93.2)/9 (5.6)/2 (1.2)	170 (83.7)/28 (13.9)/5 (2.5)	0.017
AA/AC + CC	309 (92.2)/26 (7.8)	278 (85.5)/47 (14.56)	0.009	150 (93.2)/11 (6.8)	170 (83.7)/33 (16.3)	0.009
AA + AC/CC	331 (98.8)/4 (1.2)	322 (99.1)/3 (0.9)	0.691	159 (98.8)/2 (1.2)	198 (97.5)/5 (2.5)	0.398

*p Values have been adjusted for gender and age*.

**Table 3:** **Alleles and genotypes of the ADAM10 SNPs in the cases and controls of cohorts I + II**.

	Cases *n* (%)	Controls *n* (%)	ORs (95% CI)	*p* Values	*q* Values
**rs653765 G > A**					
G/A	832 (83.8)/160 (16.4)	874 (82.8)/182 (17.2)	1.07 (0.85–1.35)	0.582	4.656
GG/GA/AA	348 (70.2)/136 (27.4)/12 (2.4)	363 (68.7)/148 (28.0)/17 (3.2)	1.07 (0.85–1.35)	0.581	4.647
GG/GA + AA	348 (70.2)/148 (29.8)	363 (68.7)/165 (31.3)	1.05 (0.80–1.38)	0.716	5.725
GG + GA/AA	484 (97.6)/12 (2.4)	511 (96.8)/17 (3.2)	1.33 (0.63–2.81)	0.459	3.673
**rs514049 A > C**					
A/C	949 (95.7)/43 (4.3)	968 (91.7)/88 (8.3)	1.97 (1.35–2.87)	0.000	0.003
AA/AC/CC	459 (92.5)/31 (6.3)/6 (1.2)	448 (84.9)/72 (13.6)/8 (1.5)	2.01 (1.29–3.11)	0.002	0.016
AA/AC + CC	459 (92.5)/37 (7.5)	448 (84.8)/80 (15.2)	2.18 (1.44–3.29)	0.000	0.002
AA + AC/CC	490 (98.8)/6 (1.2)	520 (98.5)/8 (1.5)	1.24 (0.43–3.59)	0.697	5.576

*ORs and p values have been adjusted for gender and age; q values were obtained using Bonferroni correction*.

**Figure 1:**
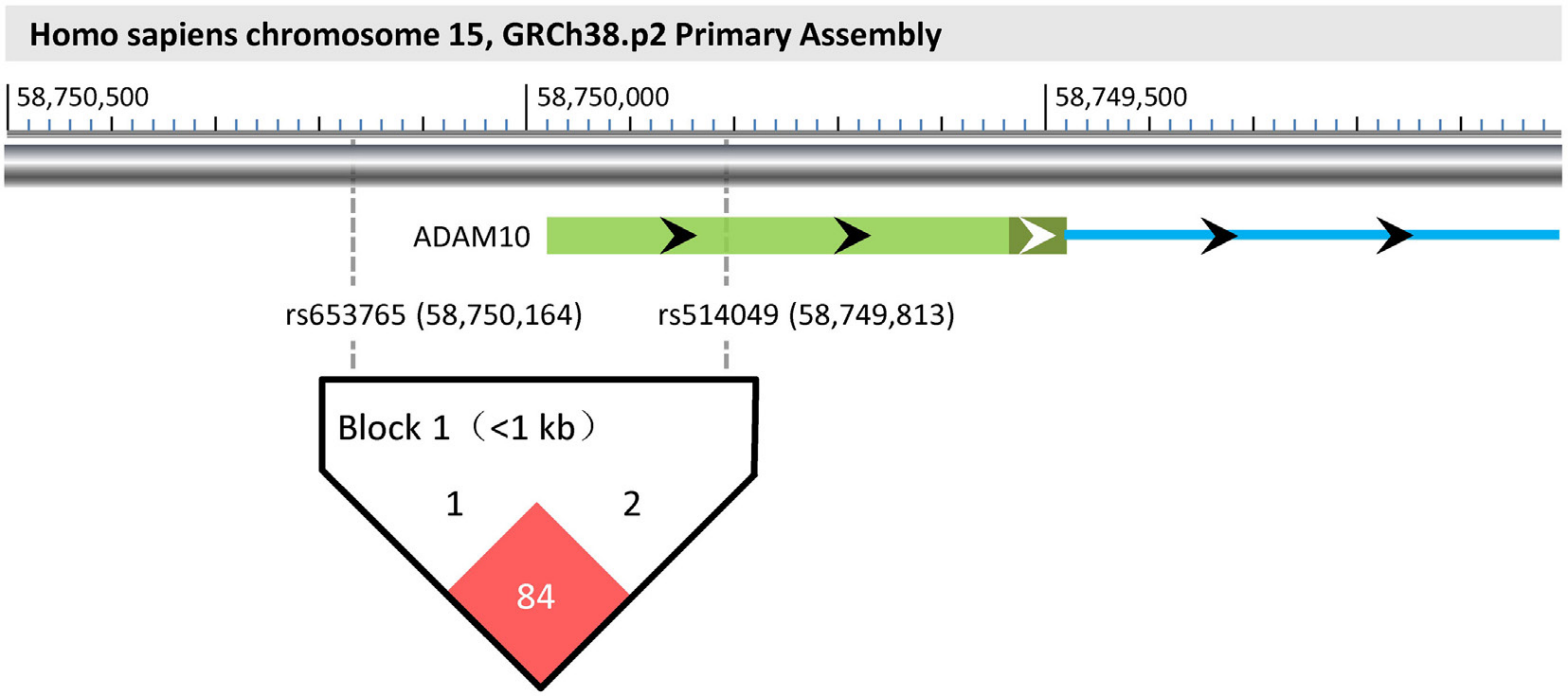
**Haplotype block (rs653765–rs514049) and its functional implications**. According to the GRCh38.p2 primary assembly, ADAM10 is encoded in *Homo sapiens* chromosome 15 (58,595,204–58,749,978). Its first exon and an upstream part of the first intron are individually shown as a light/dark green bar and a blue line, respectively. The light part of the green bar represents the 5′UTR of the ADAM10 gene. In the visual, rs653765 and rs514049 are located upstream of the transcription start site (−186 bp) and the 5′UTR of the ADAM10 gene, respectively. Using Haploview 4.2, a haplotype block (rs653765–rs514049, *D*′ value = 0.84) was further constructed in this study, implying its functional implications with TLE through influencing promoter activities of the ADAM10 gene.

As shown in Figure 1, a haplotype block (rs653765–rs514049, *D*′ value = 0.84) was successfully constructed in this study, which might influence promoter activities of the ADAM10 gene due to its particular locus. The frequencies of the ADAM10 haplotypes were then compared between the cases and controls in cohorts I + II. As a result, the case ratio of the AC haplotype was demonstrated to be lower than the control ratio (Table 4, 4.3 vs. 6.8%, *p* = 0.013), indicating that the AC haplotype likely exerts protective effects against TLE by weakening the transcriptional activities of the ADAM10 gene.

**Table 4:** **The ADAM10 haplotypes in the cases and controls of cohorts I + II**.

	Haplotypes	Frequency ratios (%)	Case ratios (%)	Control ratios (%)	*p* Values
rs653765–rs514049	GA	82.5	83.8	81.2	0.124
	AA	11.1	11.9	10.4	0.311
	AC	5.6	4.3	6.8	0.013

### Impacts of the ADAM10 SNPs on Age at Onset of TLE

**Figure 2:**
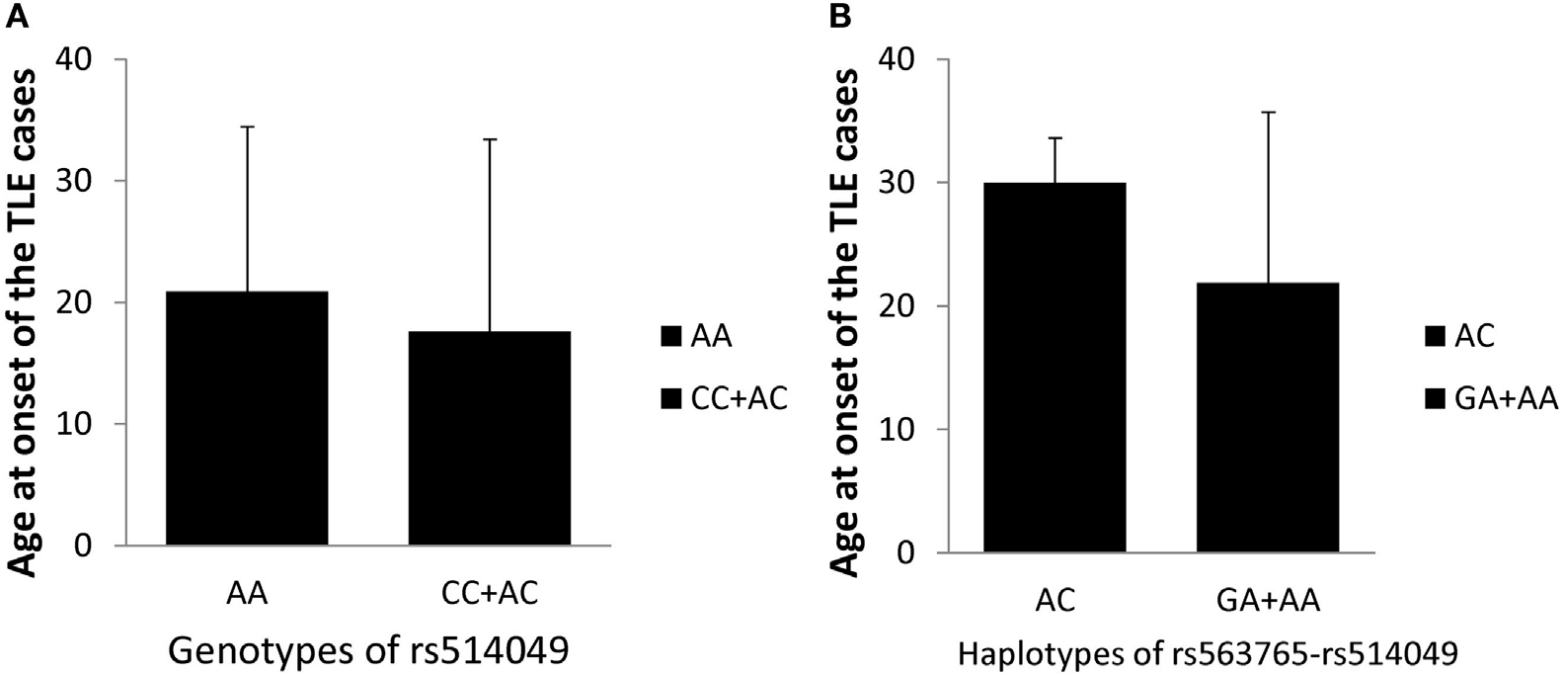
**Impacts of the AA genotype (A) and AC haplotype (B) on the age at onset of TLE cases in cohorts I + II**.

Based on the positive findings above, this study further evaluated the impacts of the AA genotype and AC haplotype on the age at onset of TLE patients in cohorts I + II. However, as shown in Figure 2, no significant differences in age at onset were observed between the AA genotype (*n* = 459, 21 ± 14 years) and the AC + CC genotypes (*n* = 37, 19 ± 16 years) or between the AC haplotype (*n* = 6, 30 ± 3 years) and the GA + AA haplotypes (*n* = 354, 22 ± 14 years) after adjustments for gender and age (*p* = 0.454 and *p* = 0.392, respectively). These findings indicated no association of the ADAM10 SNPs with age at onset of TLE.

### Impacts of the ADAM10 SNPs on Initial Seizure Types of TLE

**Figure 3:**
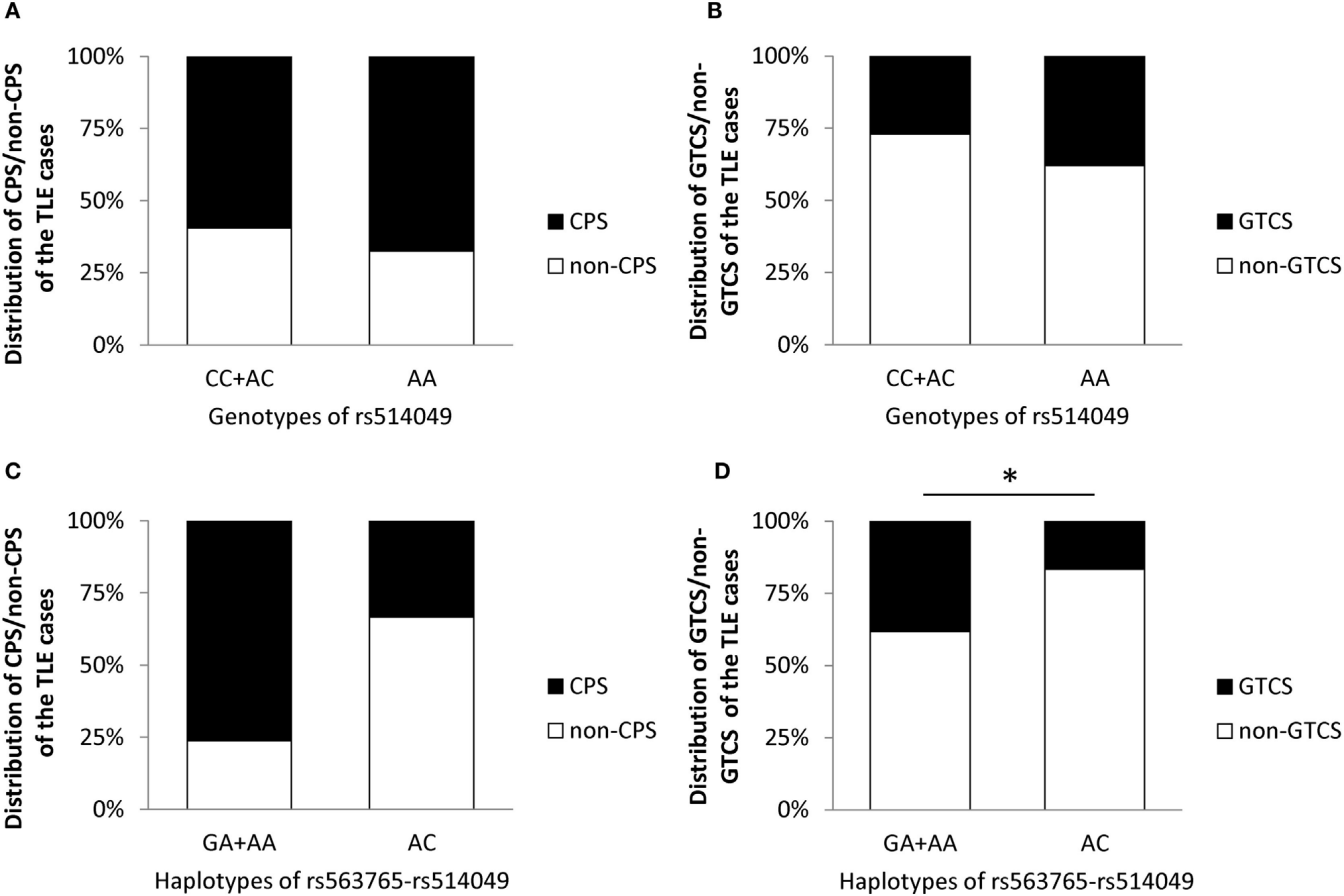
**Impacts of the AA genotype (A,B) and AC haplotype (C,D) on initial seizure types of the TLE cases before treatment in cohorts I + II**. * represents adjusted *p* < 0.05.

Similar to age at onset, this study also explored the impacts of the AA genotype and AC haplotype on the initial seizure types of the TLE patients before treatment in cohorts I + II. As shown in Figure 3, the incidence of GTCS was significantly lower in carriers of the AC haplotype than the GA + AA haplotypes after adjusting for gender and age (*p* = 0.019), indicating the potential effects of the AC haplotype against GTCS. However, no significant differences in CPS were seen between the AC haplotype and the GA + AA haplotypes (*p* = 0.997) or in initial seizure types (CPS and GTCS) between the AA and AC + CC genotypes (*p* = 0.301 and *p* = 0.897, respectively).

### Impacts of the ADAM10 SNPs on Response to Drug Treatment of TLE

**Figure 4:**
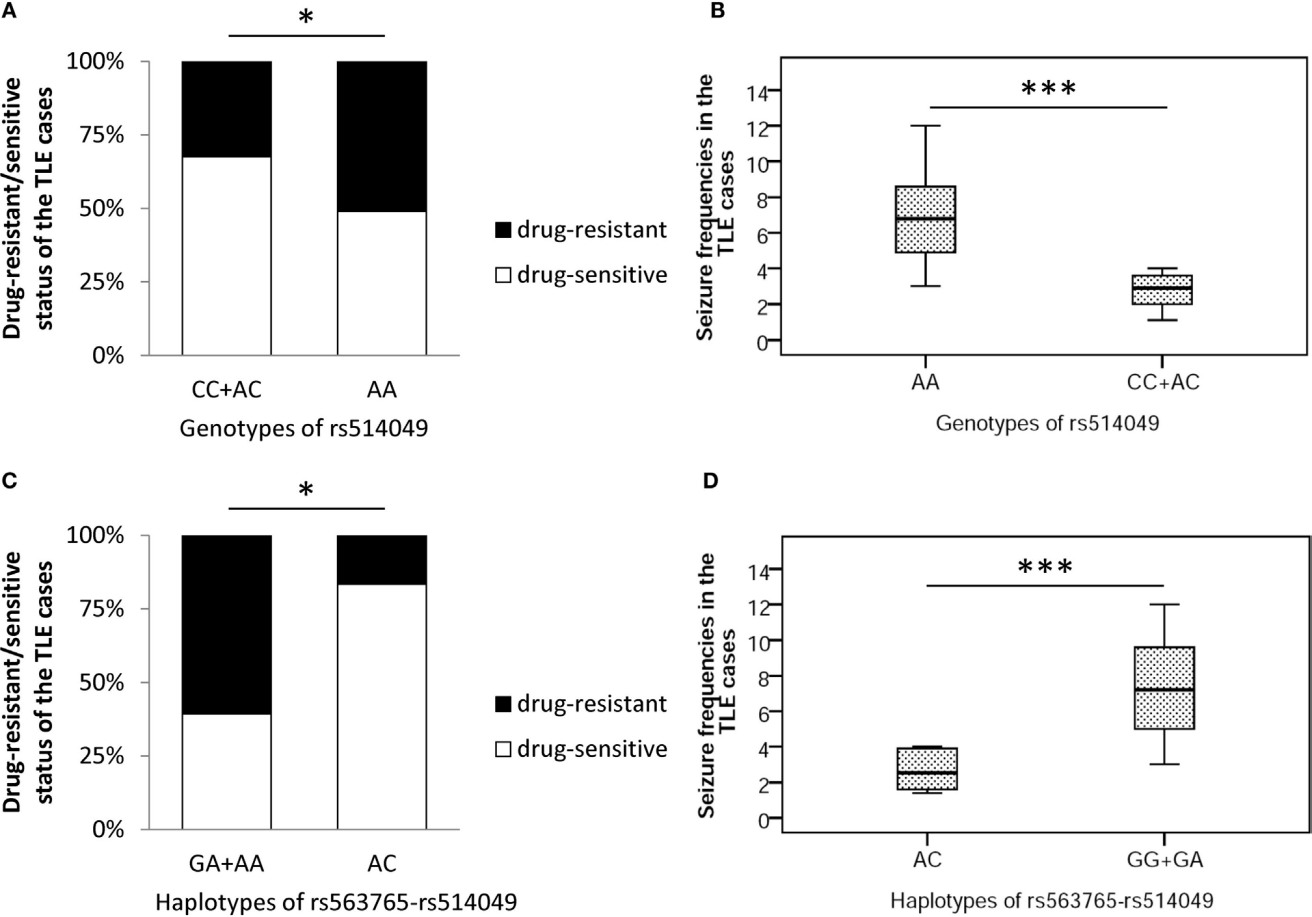
**Impacts of the AA genotype (A,B) and AC haplotype (C,D) on response to drug treatment of TLE cases in cohorts I + II**. * and *** represent adjusted *p* < 0.05 and *p* < 0.001, respectively.

Finally, this study investigated the impacts of the AA genotype and AC haplotype on responses to drug treatment of TLE patients in cohorts I + II. As shown in Figure 4, the incidences of drug-resistant cases and seizure frequencies were significantly higher in carriers of the AA genotype than the CC + AC genotypes (*p* = 0.017 and *p* = 0.000, respectively) but were decreased in carriers of the AC haplotype compared with the GA + AA haplotypes (*p* = 0.038 and 0.015, respectively) after adjusting for gender, age, and age at onset. These findings strongly imply that the AA genotype and AC haplotype function in response to TLE drug treatments.

## Discussion

Based on the protective effects of ADAM10 against epileptic seizures, this study was designed to investigate the association between ADAM10 and TLE from a genetic perspective. As expected, we first observed the roles of the promoter variants in TLE: the A allele and the AA genotype at rs514049 were associated with predisposition to TLE, whereas the AC haplotype (rs653765–rs514049) exerted protective effects against TLE. Further analyses of the TLE patients indicated that the AA genotype functioned as a predisposing factor to drug-resistant TLE and the AC haplotype as a protective factor against GTCS and drug-resistant TLE.

Temporal lobe epilepsy is the most common form of partial epilepsy in adolescents and adults. It is estimated that TLE represents almost half of all epileptic seizures in adults (21). Similarly, the age at onset fluctuated by a wide margin in this study, possibly because TLE is a disease with different genetic and environmental factors. To some extent, early-onset TLE in adolescents usually originates from autosomal dominant inheritance and inborn errors, such as familial mesial TLE, familial lateral TLE, malformation of cortical development, and neonatal hypoxic ischemic encephalopathy (22, 23). However, the etiologies of late-onset TLE in adults are relatively complex and remain elusive. In recent years, the coexistence of AD and epileptic seizures has raised a great deal of concern (3–8). AD, a typical aging disease, shows a higher prevalence of epileptic seizures than the general population. In particular, as a key modulator against the deposition of Aβ in AD, ADAM10 protein plays a protective role against epileptic activities (11–14). Thus, we hypothesized an association between the ADAM10 SNPs and age at onset of TLE. In this study, no association was observed between the ADAM10 promoter variants and age at onset of TLE. However, a genetic observation is not sufficient to deny the potential role of ADAM10 protein in late-onset TLE, which still deserves to be evaluated in future research.

The symptoms of TLE are highly complex, including, but not limited to, indescribable auras, automatisms, and autonomic phenomena (24). In clinical practice, they are often simplified as three seizure types: simple partial seizures (SPS), CPS, and GTCS. In comparison with localized SPS within temporal lobes, CPS and GTCS are more serious due to their additional involvements in disturbance of consciousness and secondary generation, respectively. In light of anatomical location, CPS partly results from transient interception of the bilateral ascending reticular activating system (ARAS). GTCS is implicated in aberrant discharges of motor function areas in frontal lobes. CPS and GTCS do not represent the same activated networks; therefore, recognizing seizure types is necessary to locate seizures and unveil their pathogenic networks. Notably, the deposition of neurotoxic Aβ was found in frontal lobes (25–27) as well as temporal lobes, which implies that ADAM10 might also function in frontal lobes due to its negative regulation on Aβ. In this study, the incidence of GTCS was observed to be significantly lower in carriers of the AC haplotype, which further supports the involvement of ADAM10 in frontal lobes. In addition, considering the role of the AC haplotype in decreasing transcriptional activities of the ADAM10 gene (17), it should be a functional variant for protection against GTCS.

Despite the development of a large number of new-generation antiepileptic drugs in recent decades, DRE still accounts for approximately one-third of epileptic cases (28). Notably, DRE is merely a clinical syndrome in light of the definition proposed by the ILAE (20), and its main source is TLE. Currently, several hypotheses are thought to explain drug-resistant TLE, including transporter abnormalities (involved in overexpression of P-glycoprotein, multiple drug resistance 1, and multidrug resistance protein), loss of drug sensitivity, and seizures beget seizures by a cascade of events, such as neuronal damage, sprouting of neuronal axons, and new synapse formation (29–34). Interestingly, the levels of Aβ were recently observed to increase in the temporal cortex and hippocampus of drug-resistant patients, but no extracellular amyloid plaques were identified. This finding suggested that abnormal Aβ may function in the pathogenesis of drug-resistant TLE, but not in the same manner as AD (35). Based on these findings, we speculate that downregulated ADAM10 might play a pathological role in drug-resistant TLE through enhancing expression of Aβ. In the case–control study, we first demonstrated the association of ADAM10 with drug-resistant TLE from the genetic perspective. Considering that the AC haplotype reduced transcriptional activities of the ADAM10 promoter (17), these findings strongly support the notion that the AA genotype and AC haplotype function as factors of predisposition to/protection against drug-resistant TLE, respectively.

Several limitations should be acknowledged in the study. First, the relationship between the expression of ADAM10 in epileptogenic foci and its promoter variants remains undetermined because almost all enrolled cases accepted drug treatments; thus, it is difficult to obtain surgical brain samples for further experiments. Second, a short evaluation time (1 year) was used to identify patients with drug-resistant TLE; however, we agree with Schiller and Najjar that drug resistance is a graded process (36). Longer evaluation cycles (≥2 years) would likely bring about different cohorts than the drug-resistant TLE cohort used in this study; thus, the susceptibility to/protection against drug-resistant TLE in this study needs to be extended with caution under the condition of other evaluation times. Third, all enrolled subjects were of Han Chinese descent, and the minor allele frequencies at rs653765/rs514049 (0.17/0.07) are different from those in other populations, according to the international HapMap project, such as Utah residents of Northern and Western European ancestry (0.30/0.36) and African ancestry in the Southwestern United States (0.26/0.17). Thus, caution should be exercised before generalizing these findings to other ethnic populations.

In summary, this study is the first to demonstrate the association of the ADAM10 promoter variants (the A allele and AA genotype/the AC haplotype) with predisposition to/protection against TLE, respectively. In particular, the AA genotype and the AC haplotype show their effects upon GTCS and drug-resistant TLE.

## Author Contributions

HT, JZ, and XZ undertook data analyses and wrote the paper with all drawings in Figures 1–4; HT, ZM, Ying C, FS, and LY carried out subject enrollments and specimen collection; JZ and XZ carried out genotyping; LC, HZ, Yujie C, Yanyan C, and SZ carried out DNA extraction; and BZ and KL designed the study. All authors discussed the results and reviewed the paper.

## Conflict of Interest Statement

The authors declare that the research was conducted in the absence of any commercial or financial relationships that could be construed as a potential conflict of interest.
